# Rationale and opportunities in estimating the economic burden of seasonal influenza across countries using a standardized WHO tool and manual

**DOI:** 10.1111/irv.12491

**Published:** 2017-11-16

**Authors:** Nathorn Chaiyakunapruk, Surachai Kotirum, Anthony T. Newall, Philipp Lambach, Raymond C. W. Hutubessy

**Affiliations:** ^1^ School of Pharmacy Monash University Malaysia Selangor Malaysia; ^2^ Department of Pharmacy Practice Faculty of Pharmaceutical Sciences Center of Pharmaceutical Outcomes Research (CPOR) Naresuan University Phitsanulok Thailand; ^3^ School of Pharmacy University of Wisconsin Madison WI USA; ^4^ Asian Centre for Evidence Synthesis in Population Implementation and Clinical Outcomes (PICO) Health and Well‐being Cluster, Global Asia in the 21st Century (GA21) Platform, Monash University Malaysia, Bandar Sunway Selangor Malaysia; ^5^ Social and Administrative Pharmacy Department Faculty of Pharmacy Rangsit University Muang, Pathumthani Thailand; ^6^ School of Public Health and Community Medicine University of New South Wales Sydney NSW Australia; ^7^ Initiative for Vaccine Research World Health Organization Geneva Switzerland

**Keywords:** cost of illness, economic burden, estimation, manual, seasonal influenza

## Abstract

Influenza disease burden is recognized as one of the major public health problems globally. Much less is known about the economic burden of influenza especially in low‐ and middle‐income countries (LMICs). A recent systematic review on the economic burden of influenza in LMICs suggests that information is scarce and/or incomplete and that there is a lack of standardized approaches for cost evaluations in LMICs. WHO commissioned and publicized a *Manual for estimating the economic burden of seasonal influenza* to support the standardization of estimates of the economic burden of seasonal influenza across countries. This article aims to describe the rationale of this manual development and opportunities that lie in collecting data to help policymakers estimate the economic burden of seasonal influenza. It describes a manual developed by WHO to help such estimation and also links to relevant literature and tools to ensure robustness of applied methods to assess the economic burden associated with seasonal influenza, including direct medical costs, direct non‐medical costs and indirect costs.

## 1 INTRODUCTION

In 2010, the World Health Organization (WHO) commissioned the development of a manual for estimating the disease burden associated with seasonal influenza.^1^ A recent systematic review on the economic burden of influenza in low‐ and middle‐income countries (LMICs) suggests that information is scarce and/or incomplete and that there is a lack of standardized approaches for cost evaluations in LMICs.^2^ Given the current lack of economic burden estimates of seasonal influenza from these countries,^3^ WHO commissioned the development of this *Manual for estimating the economic burden of seasonal influenza* to support the standardization of estimates of the economic burden of seasonal influenza across countries.^4^

National governments require data on the economic burden of influenza disease in their countries to make informed and evidence‐based decisions to allocate limited resources optimally and to prioritize interventions in the health sector. This article describes the *Manual for estimating the economic burden of seasonal influenza which* aims to assist country officials to perform studies assessing the economic burden of seasonal influenza disease in LMICs. Such information is crucial to support decision‐making on the introduction of a influenza vaccine, complementary vaccination strategies and/or expanding vaccination target groups.

The *Manual for estimating the economic burden of seasonal influenza* has been developed primarily for use in LMICs. It provides step‐by‐step approaches on how to estimate the economic burden associated with seasonal influenza, including direct medical costs, direct non‐medical costs and indirect costs. It is a companion to other key WHO documents specific to this disease, namely *‘A manual for estimating disease burden associated with seasonal influenza* 1 and *Guidance on the economic evaluation of influenza vaccination’*.^4^ The disease burden estimated in accordance with the manual^1^ is considered crucial information and used as part of the economic burden calculation. The *WHO guide to identifying the economic consequences of disease and injury* 5 further helps to form the methodological approach used to provide the specific advice on estimation of the economic burden of seasonal influenza. The manual is complemented by existing WHO guidance on introducing new vaccines into vaccination schedules.^6^, ^7^

The *Manual for estimating the economic burden of seasonal influenza* is currently available and accessible on the WHO website (http://apps.who.int/iris/bitstream/10665/250085/1/WHO-IVB-16.04-eng.pdf). This article aims to describe the rationale of this manual development and opportunities that lie in collecting data to help policy makers estimate the economic burden of seasonal influenza. In addition, it aimed to introduce the manual to the users by summarizing key contents of the manual. Our article consists of 5 main sections. First, we describe disease burden estimation of seasonal influenza which provides a foundation of data for economic burden estimates. Second, we reviewed the general approach of economic burden estimation and summarized the specification of the economic burden estimation for influenza. Third, the whole process of economic burden estimation is briefly reported. Fourth, we suggested the analysis and presentation approach for economic burden. Last, we provided the overall conclusions of the approach to estimating the economic burden of influenza.

## 2 DISEASE BURDEN ESTIMATION OF SEASONAL INFLUENZA

WHO^1^ suggests sentinel surveillance of influenza‐like illness (ILI) to estimate mild disease outcomes, and severe acute respiratory infection (SARI) to estimate severe outcomes of disease. Surveillance of both these diagnoses provides an approximate understanding of influenza incidence with the use of data from several influenza sentinel sites.^13^ A detailed description of ILI and SARI cases and methods to estimate these outcomes can be found in *A manual for estimating disease burden associated with seasonal influenza*.^1^ A brief summary is provided below.

WHO also suggests capturing the disease incidence associated with SARI in terms of both morbidity and mortality.^1^ Measuring morbidity of laboratory‐confirmed influenza‐associated SARI cases requires data on the incidence rate, which is the number of new influenza‐associated SARI cases from the population at risk of experiencing the event in the catchment area over a defined period of time. Data that are required for disease incidence estimation can be obtained from SARI sentinel sites with known or estimable catchment populations or from appropriate hospitals—that is, hospitals that are not designated as sentinel surveillance sites but that can conduct a laboratory influenza virus test and which are large hospitals with good electronic data coding systems; routinely test for influenza virus among eligible patients; record data consistently and completely. Mortality from SARI is estimated using an in‐hospital case fatality ratio (CFR). To provide a CFR with reasonable precision, a large number of individuals must be followed as the CFR for influenza, including influenza‐associated SARI, is relatively low. However, WHO suggests limiting in‐hospital CFR data to only those SARI cases confirmed for influenza. If data are available from multiple sentinel sites, the incidence of SARI should be pooled so long as the case definition is the same, the sentinel sites are well distributed and the catchment area is representative of the country.

To describe the magnitude of disease in a target area (eg, a province, state, region or country level), the estimated number of cases of a defined catchment area can be used. Based on incidence rates (for either influenza‐associated ILI or SARI) in the catchment area, we can deduce the national incidence rate for a given outcome by multiplying the rates of the catchment area with the total national population (these multipliers should be used for smaller age groups, and separately for ILI and SARI). The estimated national incidence rate is crucial information for deriving the economic burden of influenza disease. Using the average of data from multiple influenza seasons helps one to account for year‐by‐year variation in incidence and severity, with a minimum of 3 years of surveillance data being recommended by WHO.^1^

These approaches have some practical limitations. ILI sentinel surveillance sites in most cases may not have a known population denominator. The percentage of confirmed case among those tested ILI cases is used to calculate the total number of actual ILI cases. Cases of SARI require laboratory‐confirmed influenza testing by polymerase chain reaction (PCR), which is resource intensive.

## 3 THE OVERALL APPROACH OF ECONOMIC BURDEN ESTIMATION

Economic burden is defined by the direct and indirect cost of an illness due to a disease or an injury.^5^ Estimates of economic burden capture the economic impact of an illness of interest both within the health sector and outside of the health sectors as well as at both the microeconomic and macroeconomic levels.

Although seasonal influenza can have broader economic impacts (eg, on long‐term medical costs, long‐term productivity or national economic growth), for practical reasons this manual deals with only direct and indirect costs.^5^ Direct costs are the costs associated with treatment of an illness or disease. These costs generally include direct medical and direct non‐medical costs. Direct medical costs are the costs related to treatment incurred both within and outside health facilities—that is, costs of ambulatory (outpatient) care, hospitalization, pharmaceuticals and other consumable costs (eg, self‐treatment). Direct non‐medical costs are illness‐related expenditures that do not relate directly to medical treatment (eg, transportation costs to hospital, additional food costs and extra expense for accommodation). Indirect costs are defined as the value of lost production because of reduced working time (for both patients and caregivers) during the episode illness or while receiving health care (ie, treatment for influenza). These costs are called productivity losses/costs resulting from the illness.

Economic burden can be estimated through two main approaches. The first is the prevalence‐based approach which is defined by the WHO^3^ as an assessment of economic consequences of a disease or a group of diseases from a cross‐sectional point of view. Influenza infection is often short‐lived, and the number of cases with symptoms in a population can vary over time. Hence, the number of cases at any one time is not a reliable indicator of the economic burden of the disease. The prevalence‐based approach is therefore usually not suitable for ascertaining the total economic burden of seasonal influenza. The second approach is the incidence‐based approach which includes only new cases over a specified period. This approach is useful for ascertaining the impact of a disease longitudinally so that one can understand the whole impact of the disease over the specified period of time.

A previous systematic literature review of economic burden and economic evaluation of seasonal influenza found a total of 140 studies worldwide of which 39 studies (28%) were cost‐of‐illness studies.^8^ Two articles did not state their scope (ie, setting) or perspective as would usually be done in a proper reporting of cost studies.^9^ Thirty‐two (82%) of the 39 studies were conducted in high‐income countries. Societal perspective—that is, an analysis that includes all costs and benefits of a health intervention regardless of who is paying for it—was commonly used (21 studies, 54%). Among the 24 studies reporting a time horizon, 15 studies specified it as a 1‐year time horizon. Nine studies quantified direct medical, direct non‐medical and indirect costs, while 8 studies included only direct medical and direct non‐medical costs. Fourteen studies quantified indirect costs. Seventeen studies estimated economic burden on the basis of laboratory‐confirmed seasonal influenza cases, while 21 studies estimated it using clinically diagnosed cases only. A recent systematic review of economic evaluation of influenza vaccines in LMICs stated that most economic data used non‐laboratory‐confirmed SARI or ILI. Indirect costs were not used in these analyses.^10^ Finally, no study has taken into account the costs of informal care among non‐medically attended care (ie, costs incurred by people who do not seek care at formal health facilities).

Approaches to economic burden analysis can vary from study to study. To help standardize the approaches for the economic burden of influenza, Table 1 provides the suggested key specifications for an estimation approach. Accordingly, the economic burden of influenza should be calculated using estimates of (severe and non‐severe) seasonal influenza cases among the general population derived based on the manual for estimating disease burden associated with seasonal influenza.^1^ However, the disease burden can also be used to estimate the burden among specific risk groups such as pregnant women or people with chronic disease. A societal perspective should be undertaken for economic burden estimation.^5^, ^11^ Therefore, cost estimates should include direct medical, direct non‐medical and indirect costs for both households and the health system. The WHO disease burden manual suggests capturing influenza‐associated SARI and ILI cases with laboratory confirmation. The incidence of these cases is used to estimate the total number of ill cases among the target population. Previous guideline for estimating the economic burden of diarrhoea disease recommends estimating non‐medical provider care costs—that is, costs associated with self‐care or over‐the‐counter medicines.^12^ Such estimation is not included in the base case analysis of this approach as it may not always be feasible for seasonal influenza because of the high cost of data collection and challenges in identifying individuals not seeking medical provider care. Informal care costs among non‐medically attended care associated with seasonal influenza should, however, be included in the sensitivity analysis if data collection is feasible. As seasonal influenza is an acute disease with a relatively short duration (in days and weeks rather than years) and insignificant long‐term sequelae, discounting is not applied for economic burden estimation.

**Table 1:** Specifications for estimation of the economic burden

Methodological issues	Specification for influenza	Justification
Seasonal influenza burden	Laboratory‐confirmed	Based on WHO disease burden estimation
Burden estimation approach^a^	Incidence‐based approach	Based on WHO guideline for quantifying economic burden^5^
Perspective	Societal	Based on WHO guideline for quantifying economic burden^5^
Time horizon^b^	1 year (maximum)^c^	Illness symptoms span days to weeks. Capturing the consequences of influenza with 1‐year time horizon would be sufficient.
Discounting^d^	No discounting required	Time horizon is less than 1 year; there is no need for discounting for this estimation.
Informal care cost among non‐medically attended care	Not covered in base case analysis	Informal care cost among non‐medically attended care is optional for data collection or for including in a sensitivity analysis
Premature mortality cost	Not captured	Much less contribution to economic burden of seasonal influenza

a Situation scenario for which the current burden of a disease can be calculated.

b Time horizon is a period that needs to cover all relevant resource use under a cost study.

c To capture seasonality, at least 3 years of surveillance data is recommended according to the disease burden manual. The economic burden can then be estimated using such information that takes year‐by‐year variability into account.

d Discounting calculates the present value of costs and consequences occurring in the future.^16^

## 4 OVERALL PROCESS OF ECONOMIC BURDEN ESTIMATION FOR SEASONAL INFLUENZA

To describe the overall process for estimating the economic burden of seasonal influenza, we suggest a 7‐step process, which can be categorized into 3 main activities: identification, data collection and measurement, and valuation (Table 2).^14^

**Table 2:** Process for estimating the economic burden of influenza illness

Step	Process	Details
1	Identification of required resources	All resources used in the influenza episode
2	Planning the sampling frame and data collection	Overall planning for data‐gathering
3	Measuring hospitalization resource utilization	Direct medical cost
4	Measuring ambulatory care resource utilization	
5	Determining unit costs	
6	Estimating out‐of‐pocket (informal care costs among medically attended care) and indirect costs (including copayment/self‐aids/community care)	Direct medical and direct non‐medical costs and indirect cost
7	Informal care costs among non‐medically attended care	Direct medical, direct non‐medical and indirect cost

### 4.1 Step 1: identification of required resources

The first step is to identify all resources used in the episode of seasonal influenza. These should include the quantity or frequency of the following: medications, medical supplies, diagnostic tests, hospital bed‐days, outpatient visits, travel, hours or days absent from work or productivity losses of both patients and caregivers, and informal care visits. Ideally, all costs should be included in the full analysis, but a partial analysis can be performed on the basis of data availability—especially non‐medical provider care costs.

### 4.2 Step 2: planning the sampling frame and data collection

The second step is to plan the sampling frame and data collection appropriate to each country. As the goal of the study is to estimate a country's economic burden associated with seasonal influenza from a societal perspective, it is important to determine the settings so that data collection is representative of the province, state, region or country. The WHO manual for estimating disease burden associated with seasonal influenza recommends that specific sentinel surveillance sites should be identified which have captured laboratory‐confirmed data on SARI and ILI. These facilities used for inpatient and outpatient data collection should be representative of the target area (province, state, region or country) for which estimation is being evaluated. Preferably, these sites should be a random sample of locations or a selection of locations that represent that target level.

In addition to selection of facilities, decisions on the number sites and the sample sizes within each site will need to be made depending on how precise the estimates need to be and the resources available for collection of data. It is also crucial to determine the subjects by random selection unless the whole population is included, as shown in a previous study of inpatient visits.^15^ More information on how to collect samples for estimating disease burden can be found in A manual for estimating disease burden associated with seasonal influenza.^1^ It would be advantageous if samples for collecting data on the quantity of resource utilization associated with the treatment would be selected in a similar manner to those for estimating disease burden as the confirmed SARI and ILI cases can be identified from specific sentinel surveillance sites.

### 4.3 Step 3: measuring hospitalization resource utilization and Step 4: measuring ambulatory care resource utilization

The third and fourth steps involve measuring the use of resources associated with the treatment^16^ of laboratory‐confirmed SARI hospitalization and ILI outpatient visits. To estimate resource utilization for hospitalizations of seasonal influenza‐associated SARI patients, resources utilized during the whole hospitalization episode should ideally be included and comprehensively collected. The resource utilization data to be collected include length of stay, the type of setting (ie, intensive care unit or general medicine), frequency and amount of laboratory work, medication, diagnostic tests, therapeutic interventions, duration and route of administration of medications (eg, antihistamines, antipyretics) and physicians’ or healthcare providers’ consultations. The approaches to estimating resource utilization for hospitalizations can be divided into 3 groups, namely (1) electronic hospital database (EHD), (2) existing literature, and (3) primary data collections. It is important to note that all included cases for approaches 1 and 3 must be laboratory‐confirmed as influenza‐associated SARI/ILI. Table 3 compares the 3 approaches in terms of advantages and disadvantages.

**Table 3:** Comparisons of approaches for measuring resource utilization

Approaches	Advantages	Disadvantages
Electronic hospital database (EHD)	Fast and convenient Less costly	EHD may not be evaluated for its reliability May not be representative of the whole country unless there are national datasets Requires technical skills for analysis
Existing literature^a^ or estimates from existing government statistics	Convenient	Might not be fully representative to the study May not fully capture all utilizations
Primary data collections	Fully capture all data required especially prospective study	Time‐consuming and costly

a Existing literature should be appraised for quality.

Use of an EHD would be the optimal approach if the database is valid and representative of national costs. In practice, it may not be possible to use an EHD in LMICs, thus limiting the gathering of quality data. Analysis of existing literature is an alternative option so long as the study findings are relevant and representative. The study must provide information on resource utilization for hospitalized influenza cases. For example, the study by Savy and colleagues used existing articles to estimate the use of resources in countries of Latin America and the Caribbean.^17^ If neither EHDs nor relevant literature are available, primary data collection remains an option. This is described in more detail in this manual as its use may be required predominantly in LMICs. The cases included in the primary data collection must be laboratory‐confirmed cases.

Data on the resources utilized during the whole disease episode involving outpatient health facilities should be included and comprehensively collected to estimate resource utilization for outpatient visits of seasonal influenza‐associated ILI patients. Outpatient care refers to all cares that do not require hospital admission. Resource utilization data to be collected include the number and type of visit, the type of department or facility (ie, internal or general medicine), frequency and amount of laboratory work, medication, diagnostic tests, therapeutic interventions and physician's consultation. The approaches to estimating resource utilization for ambulatory care visits are very similar to those for hospitalizations. The approaches can be divided into 3 groups: (i) electronic ambulatory database, (ii) existing literature and (iii) primary data collections. As stated previously for EHD, data from the electronic ambulatory database, if valid and representative for the whole country, are the optimal source of health burden information. Existing literature and primary data collections represent secondary options that depend on time and budget. In reality, these different methods could be combined, as each option alone may not cover all data requirements. For instance, a literature review might be combined with primary data collection. As the approaches are similar to those for hospitalization, the details are not repeated here. Examples of data collection forms are provided in the full publication of this WHO manual.^4^ As there is no single one‐size‐fits‐all approach to data collection, investigators should use their discretion to adapt the suggested generic data‐collection forms to their own context.

### 4.4 Step 5: determining unit costs

The fifth step is to describe how the cost is valued, providing direction on how to determine unit costs. In steps 3 and 4, estimates were made of the quantities of resources used: medications, tests, hospital days and number of visits used in the treatment of a sample of laboratory‐confirmed seasonal influenza‐infected cases. In this section, unit cost estimates are collected for each of these resources. Information on unit costs and quantities should then be combined to estimate the total cost of treatment of a seasonal influenza case.

Unit cost data can be presented in local currency. To allow for comparisons between countries, a conversion into the hypothetical currency of ‘international dollars’ may be preferable to correct for differences in a country's purchasing power. Costs in local currency units are converted to international dollars using purchasing power parity (PPP) exchange rates. A PPP exchange rate is the number of units of a country's currency required to buy the same amounts of goods and services in the domestic market as US dollars would buy in the USA. The PPP exchange rates can be found on the WHO‐CHOICE web page. However, all unit cost estimates should be presented in a consistent currency. In addition, an index year for the analysis should be selected because costs used in the analysis may not necessarily occur during the same year as the index year for which the disease burden is estimated. The cost data should be derived from the most recent index year. The disease burden and the costs should be reported separately with their index years.

Unit cost estimates must be collected for each of the following items: (i) medications, (ii) diagnostic tests, (iii) a hospital bed‐day or routine service costs (cost of managing hospitalized patients per day) for intensive care or regular wards, and (iv) outpatient visit costs (cost of ambulatory care excluding medicines, medical supplies and diagnosis costs). Different approaches can be used to estimate unit costs according to the data available, the required level of precision, and the resources available to do the study. In addition, it is possible to use unit cost estimates from the public or private sectors. If local data are unavailable, the use of data on unit costs from neighbouring countries with similar health system costs can also be considered. However, great caution is required when taking this approach as these data may not be fully representative for the setting.

### 4.5 Step 6: estimating out‐of‐pocket (informal care costs among medically attended care) and indirect costs (including copayment/self‐aids/community care)

The sixth step is to estimate out‐of‐pocket (direct medical and direct non‐medical) costs and indirect costs among medically attended cases. To gain a more complete costing picture, one can also consider including the magnitude of out‐of‐pocket expenses (direct medical costs paid for patients and/or caregivers and non‐medical costs, such as transportation to and from heath‐care facilities, childcare for dependent children during admission and recovery, and household costs to accommodate the needs of the patient). Indirect costs are defined as the value of the time lost by patients and caregivers during the episode of illness. The term also includes productivity loss associated with premature death. These are often also referred to as productivity losses related to illness or death. A systematic review of 39 economic burden studies of seasonal influenza showed that only 14 studies included indirect costs in their estimation and only 2 studies considered indirect costs associated with premature death.^18^, ^19^ Both studies revealed that the indirect costs from years of life lost accounted for only 0.5%‐0.8% of total costs. This is because, in many groups, the CFR associated with influenza is relatively low and the contribution of indirect costs from life‐years lost to the overall economic burden is small. Consequently, the estimation of indirect costs is limited only to productivity losses borne by patients and caregivers during the acute episode of seasonal influenza.

### 4.6 Step 7: informal care costs among non‐medically attended care

The last step involves the estimation of informal care costs among non‐medically attended care. Treatments in informal health facilities or by self‐medication are quite common among ILI cases. According to studies involving community surveys on ILI behaviour in some LMICs, about a third to a half of the infected cases sought health care in informal or non‐health facility sectors.^15^, ^20^ The recent systematic review^8^ indicated that there was no study estimating the economic burden on patients who are seeking care only outside medical settings. The potential reason was the challenge in data collection among those seeking care outside medical settings because capturing these data outside health facilities would be resource intensive. However, it is important to consider how/whether to estimate the proportion of people who seek care only through informal health channels; this should be decided by local stakeholders or local government through consultations.

As specified earlier, it is proposed to include informal care costs only in a sensitivity analysis. The data sources for informal care costs range from community‐based household surveys or existing national household surveys to extrapolation from such studies in other countries.

The most valid source for estimating informal care costs is the community‐based household survey. The sample population should be patients or caregivers who have recently experienced an event. Surveys should include information on type of care sought (where, from whom), transportation costs, payment for medications, tests (if any) and consultations, and time lost from paid work (lost income). However, conducting such a survey can be challenging in terms of feasibility. Several other approaches are available for deriving informal care costs through existing secondary data. The proportion of care‐seeking in the informal sector and the informal care costs per patient among ILI‐infected cases may be obtained from existing national household surveys or may be extrapolated from studies in other countries. A range of plausible estimates should be evaluated in sensitivity analyses. The potential implications of including the cost of informal care in the total national economic burden should be reported both collectively and separately.

To facilitate selection of the optimal data‐collection approaches by manual users, we have developed Tables 4 and 5 to help for guidance. The set of questions in Table 4 is intended to facilitate the manual users’ selection of a data‐collection approach. The yes/no answer to each question will lead to the specific data‐collection approaches suggested for each scenario. Table 4 shows potential questions to consider for influenza‐associated SARI data collection. These questions would then have to be repeated when considering influenza‐associated ILI data. Example of some scenarios using a ‘traffic light’ concept (green light denotes yes, while red light denotes no) are provided (Table 5), and the data‐collection approach is suggested for each scenario. For example, for scenario ‘G’, the answers to Q1 and Q1.1 were ‘Yes’ (green light), while the answers to the remaining questions were ‘No’ (red light). For resource use, an EHD should be a preferred choice. For unit cost, conducting a unit cost study or using the WHO‐CHOICE unit cost estimates is the possible option. For out‐of‐pocket and indirect costs, data need to be collected by interviewing patients and caregivers. Details of each data‐collection approach are described in steps 3‐6. It is important to note that the traffic light concept provides only guidance for planning the data‐collection approach. The decisions belong to the analysts who must make appropriate choices based on their own judgement.

**Table 4:** Examples of questions to guide the selection of a data‐collection approach^a^

Questions guiding the selection of a data‐collection approach		Yes	No
Resource use			
Q1	Is an electronic hospital database (EHD) capturing resource use of influenza‐associated SARI/ILI available? [*Depends on the scope of estimation of economic burden by specifying the catchment area, which may be at provincial, state, regional or national level*]		
Q1.1	Is the database valid and representative^b^ of the catchment area? [*Reliability and representativeness assessment can be referred to the disease burden manual* 1]		
Q2	Do previous studies estimating resource use of influenza‐associated SARI/ILI exist?		
Q2.1	Are the findings valid and representative of the catchment area?		
Unit cost			
Q3	Are previous studies estimating resource use of influenza‐associated SARI/ILI existing?		
Q3.1	Are the findings valid and representative of the target area?		
Out of‐pocket and indirect costs (for health‐seeking cases)			
Q4	Existence of previous studies estimating out‐of‐pocket/indirect costs of influenza‐associated SARI/ILI		
Q4.1	Are the findings valid and representative of the catchment area?		

a These questions need to be repeated for influenza‐associated ILI data with some modifications (eg, electronic ambulatory databases).

b The database must be assessed for its reliability and representativeness**. (**i**)** ‘Valid’ should capture most resources consumed and record such consumption accurately. Validity of the database can be based on a previous validation study. If possible, a validation study can be performed. In some situations, where the database has been used for purposes requiring an auditing process **(**ie, a database used for claims submission has been audited during the process of its use for claims), its reliability might be subjectively justified**. (**ii**)** A ‘representative’ database should include cases from the target population of interest**.** Analysts need to ascertain that the demographic and socio‐economic characteristics of the patients receiving health care at a sentinel site or hospital are largely similar to the general population in the surrounding area. If these data are not available, then analysts need to base their judgement on their qualitative, subjective assessment of the data's representativeness. For example, if the data source is a tertiary care hospital, patients receiving care at this facility may not be representative of the seasonal influenza patients in the general population in the surrounding area because these hospitals provide care to complicated cases referred from a wide area. The types of presenting illness and the distribution of risk factors may be very different from what is expected in the surrounding general population. It may be possible to compensate for this by counting only patients from the primary catchment area around the facility.

**Table 5:**
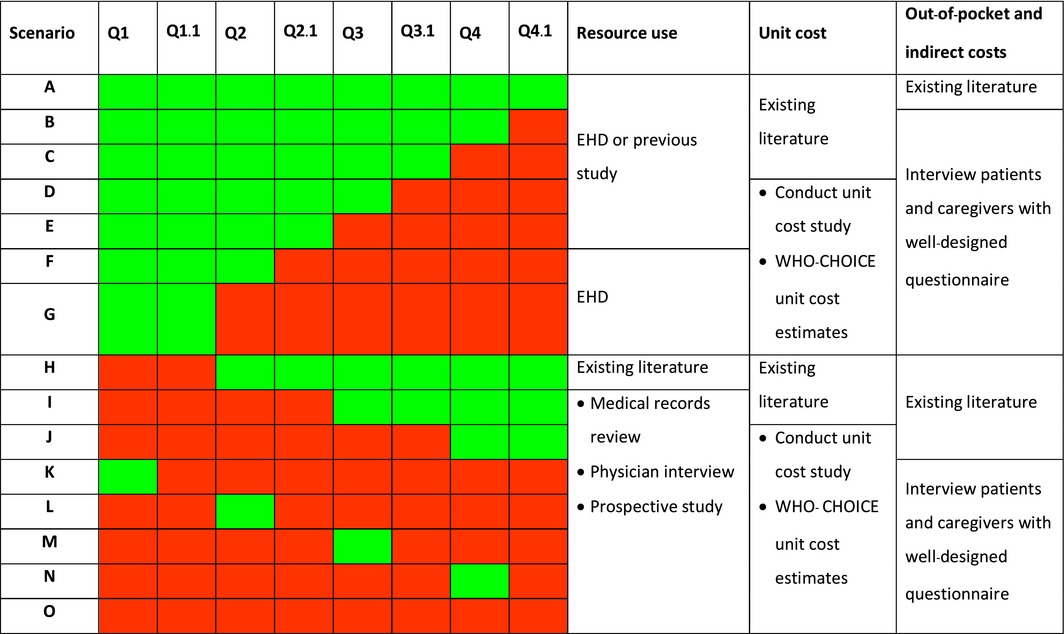
Matrices for evaluation of specific data‐collection approaches

## 5 ANALYSIS AND PRESENTATION OF THE ECONOMIC BURDEN

To calculate the relevant mean and standard deviation of patient‐specific cost data,^12^ data can be combined with disease burden estimates to derive the overall economic burden of seasonal influenza at provincial, state, regional or national levels. Results can be presented either as a specific economic burden for each year or as the overall average economic burden across multiple seasons, reflecting seasonal variations in multiple influenza season disease burden data. The age‐specific economic burden can also be analysed using an Microsoft™ Excel toolkit that accompanies this manual^4^—based on the age‐specific incidence rate from The manual for estimating disease burden associated with seasonal influenza with or without age‐specific resource use and unit cost. It is important to note that the toolkit provides a simplified example to help analysts to better understand how to calculate the economic burden after gathering relevant information. The toolkit needs to be modified by local analysts to suit each specific context.

## 6 CONCLUSION

Analysis of economic burden of seasonal influenza is an important part of the information landscape that contributes to informing evidence‐based decision‐making for the introduction or implementation of influenza vaccination programmes. Results derived from the economic burden evaluation can raise awareness among the public health and clinical communities for the burden and consequences of this important illness. More importantly, it can be used to assist for budget planning and resource allocation and can help inform input parameters for economic evaluation of prevention strategies.^4^, ^21^ A standardized approach to estimate the economic burden of influenza disease was previously unavailable. To facilitate analysis at a national level and global economic burden estimates, the manual described can help to ensure that the results of influenza economic burden estimation are valid and consistent. Its use across countries is recommended by WHO to structure global analysis of the economic burden of influenza disease and to assist countries to produce estimates to help support influenza vaccine policy decisions.

## CONFLICT OF INTEREST

None of the authors has any known competing interests.
